# Type II transglutaminase stimulates epidermal cancer stem cell epithelial-mesenchymal transition

**DOI:** 10.18632/oncotarget.3890

**Published:** 2015-05-08

**Authors:** Matthew L. Fisher, Gautam Adhikary, Wen Xu, Candace Kerr, Jeffrey W. Keillor, Richard L. Eckert

**Affiliations:** ^1^ Departments of Biochemistry and Molecular Biology, University of Maryland School of Medicine, Baltimore, Maryland, USA; ^2^ Dermatology, University of Maryland School of Medicine, Baltimore, Maryland, USA; ^3^ Reproductive Biology, University of Maryland School of Medicine, Baltimore, Maryland, USA; ^4^ Marlene and Stewart Greenebaum Cancer, University of Maryland School of Medicine, Baltimore, Maryland, USA; ^5^ Department of Chemistry, University of Ottawa, Ottawa, Ontario, Canada

**Keywords:** epidermal squamous cell carcinoma, type II transglutaminase, cancer stem cell, TG2, epithelial mesenchymal transition

## Abstract

Type II transglutaminase (TG2) is a multifunctional protein that has recently been implicated as having a role in ECS cell survival. In the present study we investigate the role of TG2 in regulating epithelial mesenchymal transition (EMT) in ECS cells. Our studies show that TG2 knockdown or treatment with TG2 inhibitor, results in a reduced EMT marker expression, and reduced cell migration and invasion. TG2 has several activities, but the most prominent are its transamidase and GTP binding activity. Analysis of a series of TG2 mutants reveals that TG2 GTP binding activity, but not the transamidase activity, is required for expression of EMT markers (Twist, Snail, Slug, vimentin, fibronectin, N-cadherin and HIF-1α), and increased ECS cell invasion and migration. This coupled with reduced expression of E-cadherin. Additional studies indicate that NF&#ξ03BA;B signaling, which has been implicated as mediating TG2 impact on EMT in breast cancer cells, is not involved in TG2 regulation of EMT in skin cancer. These studies suggest that TG2 is required for maintenance of ECS cell EMT, invasion and migration, and suggests that inhibiting TG2 GTP binding/G-protein related activity may reduce skin cancer tumor survival.

## INTRODUCTION

Epidermal squamous cell carcinoma (SCC) is among the most common cancers and the frequency is increasing at a rapid rate [1, 2]. SCC is treated by surgical excision, but the rate of recurrence approaches 10% and the recurrent tumors are aggressive and difficult to treat [2]. We propose that human epidermal cancer stem (ECS) cells survive at the site of tumor excision, that these cells give rise to tumor regrowth, and that therapies targeted to kill ECS cells constitute a viable anti-cancer strategy. An important goal in this context is identifying and inhibiting activity of key proteins that are essential for ECS cell survival. Working towards this goal, we have developed systems for propagation of human ECS cells [3]. These cells display properties of cancer stem cells including self-renew and high level expression of stem cell marker proteins [3].

In the present study we demonstrate that ECS cells express proteins characteristic of cells undergoing EMT (epithelial-mesenchymal transition). EMT is a morphogenetic process whereby epithelial cells lose epithelial properties and assume mesenchymal characteristics [4]. The epithelial cells lose cell-cell contact and polarity, and assume a mesenchymal migratory phenotype. There are three types of EMT. This first is an embryonic process, during gastrulation, when the epithelial sheet gives rise to the mesoderm [5]. The second is a growth factor and cytokine-stimulated EMT that occurs at sites of tissue injury to facilitate wound repair [6]. The third is associated with epithelial cancer cell acquisition of a mesenchymal migratory/invasive phenotype. This process mimics normal EMT, but is not as well controlled and coordinated [4, 7, 8]. A number of transcription factors (ZEB1, ZEB2, snail, slug, and twist) that are expressed during EMT suppress expression of epithelial makers, including E-cadherin, desmoplakin and claudins [4]. Snail proteins also activate expression of vimentin, fibronectin and metalloproteinases [4]. Snail factors are not present in normal epithelial cells, but are present in the tumor cells and are prognostic factors for poor survival [4].

An important goal is identifying factors that provide overarching control of EMT in cancer stem cells. In this context, several recent papers implicate type II transglutaminase (TG2) as a regulator of EMT [9–12]. TG2, the best studied transglutaminase, was isolated in 1957 from guinea pig liver extract as an enzyme involved in the covalent crosslinks proteins via formation of isopeptide bonds [13]. However, subsequent studies reveal that TG2 also serves as a scaffolding protein, regulates cell adhesion, and modulates signal transduction as a GTP binding protein that participates in G protein signaling [14]. TG2 is markedly overexpressed in cancer cells, is involved in cancer development [15–18], and has been implicated in maintaining and enhancing EMT in breast and ovarian cancer [10, 12, 19, 20]. The G protein function may have an important role in these processes [10, 21–23].

In the present manuscript we study the role of TG2 in regulating EMT in human ECS cells. Our studies show that TG2 is highly enriched in ECS cells. We further show that these cells express EMT markers and that TG2 is required to maintain EMT protein expression. TG2 knockdown, or treatment with TG2 inhibitor, reduces EMT marker expression and ECS cell survival, invasion and migration. TG2 GTP binding activity is absolutely required for maintenance of EMT protein expression and EMT-related responses. However, in contrast to breast cancer [9, 10], we show that TG2 regulation of EMT is not mediated via NFκB signaling.

## RESULTS

### TG2 is required for expression of EMT markers

**Figure 1:**
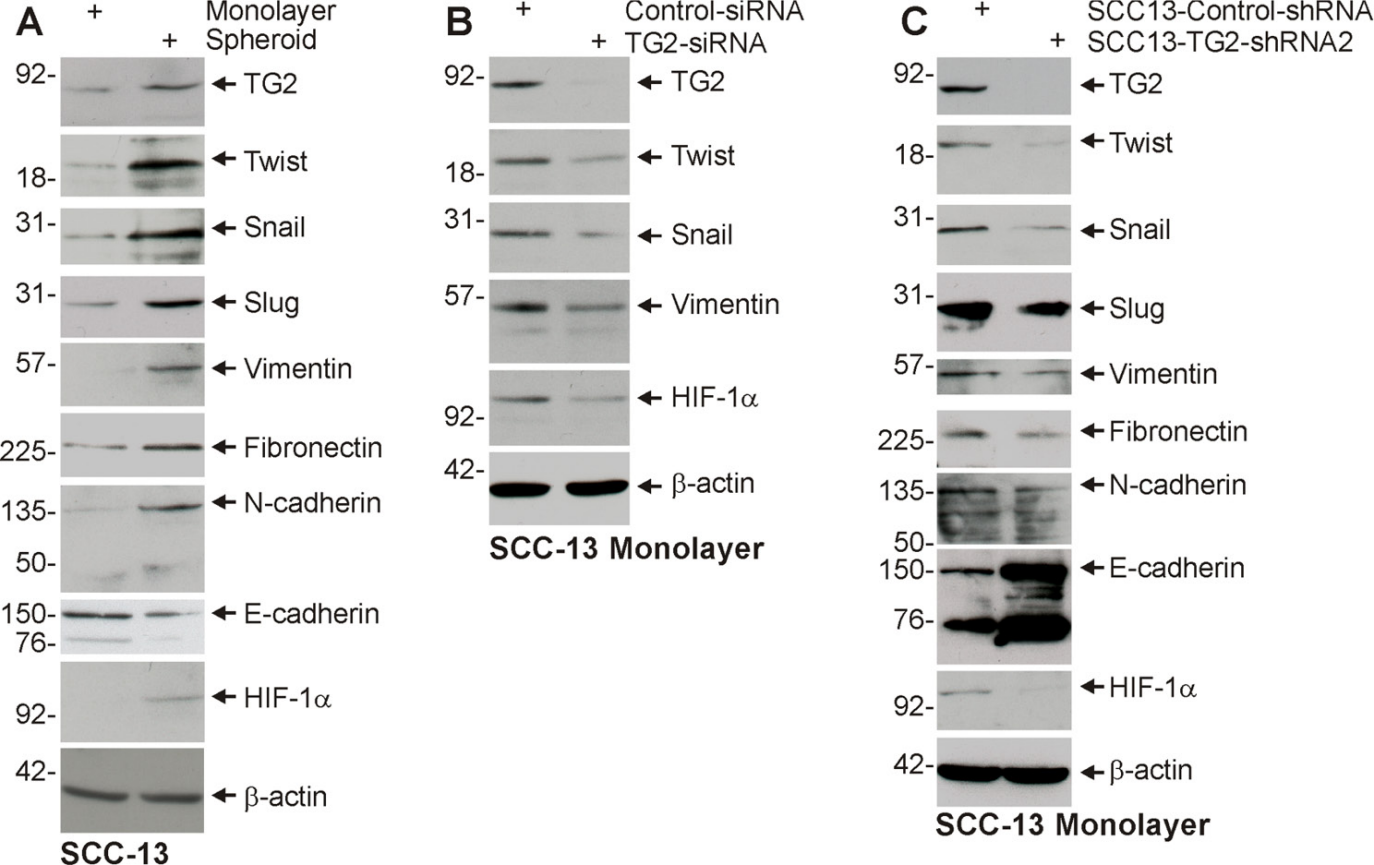
TG2 and EMT marker expression is enriched in ECS cells, and TG2 is required for EMT **A.** ECS cells have elevated TG2 and mesenchymal markers. SCC-13 cells (40,000 per well) were grown in spheroid medium in attached (monolayer) and non-attached (spheroid, ECS cells). Cells were harvested after 10 d and lysates were prepared for electrophoresis and detection of the indicated epitopes. **B.** SCC-13 cells were electroporated with control- or TG2-siRNA and cultured in spheroid medium as monolayer cultures. After 72 h extracts were prepared to assay TG2 and EMT markers. **C.** SCC13-Control-shRNA and SCC13-TG2-shRNA2 cells were grown in spheroid media for 10 d, harvested and lysates prepared for immune-detection of TG2 and EMT markers. Similar results were observed in three separate experiments.

**Figure 2:**
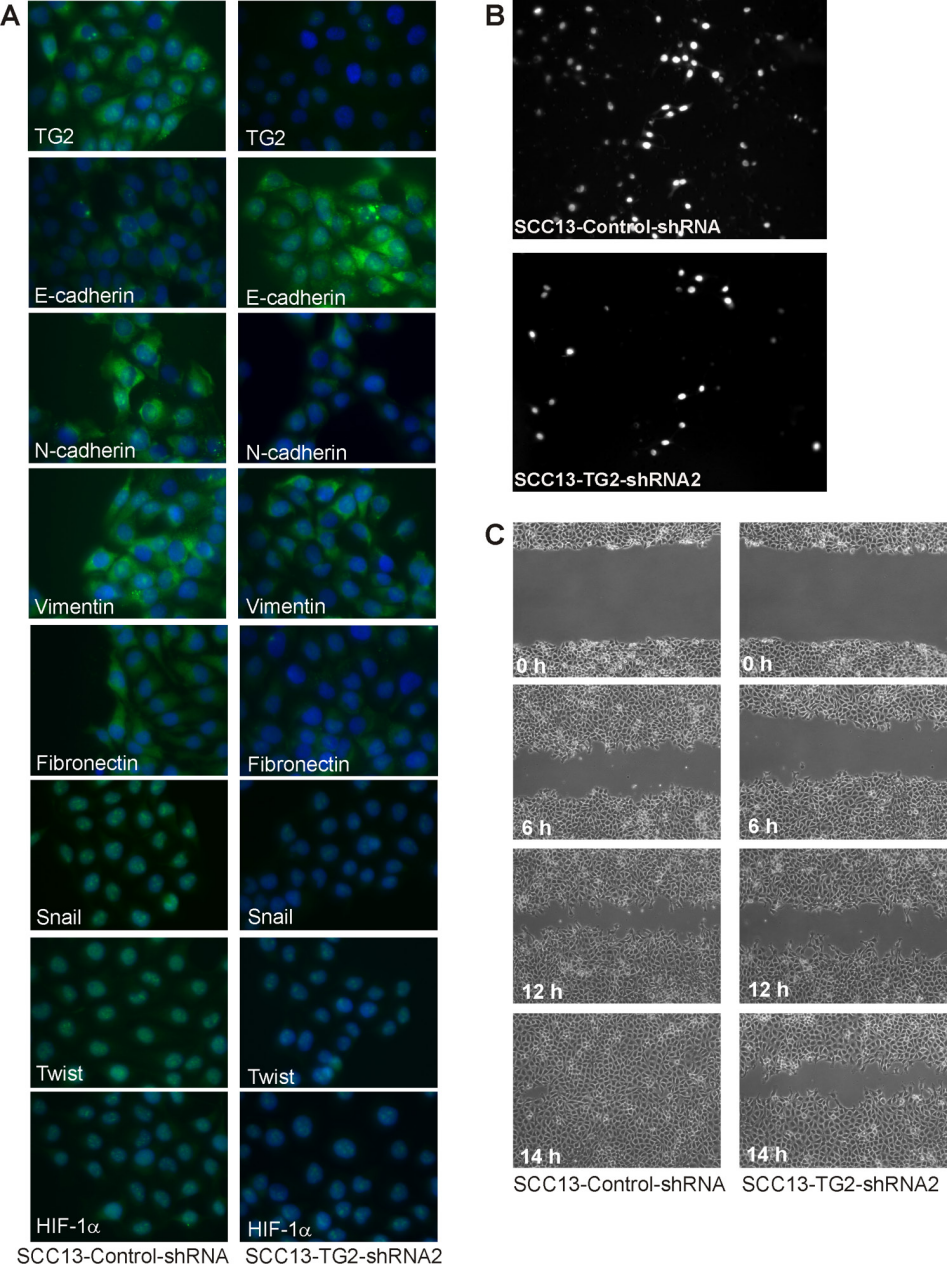
TG2 is required for EMT marker expression and invasion/migration **A.** SCC13-Control-shRNA and SCC13-TG2-shRNA2 cells were grown in spheroid medium as spheroids in non-attached culture for 8 d, and then plated as monolayers in 12 well plates (100,000 cells/well). After overnight attachment, the cells were fixed, permeabilized and incubated with antibodies specific for the indicated epitopes. **B.** SCC13-Control-shRNA and SCC13-TG2-shRNA2 cells were seeded on the upper chamber atop a matrigel-coated membrane in 1 ml of growth medium in a Millicell chamber. After migration, the membrane was removed, rinsed, fixed and DAPI stained. Nuclei were counted on the underside of the membrane using an inverted fluorescent microscope. **C.** SCC13-Control-shRNA and SCC13-TG2-shRNA2 cells (2 million) were plated on 100 mm dishes in spheroid medium in monolayer conditions. The confluent monolayers were scratched with a 10 μl pipette tip to create a wound and the released cells were removed. Images were taken at 0–14 h after the initial scratch. Similar results were observed in three experiments.

EMT is a property of tumor stem cells that confers an ability to migrate and invade surrounding tissue [24–26]. We first examined whether ECS cells express EMT markers. Non-stem cancer cells and ECS cells, derived from the SCC-13 cancer cell line, were analyzed for expression of EMT markers. Fig. 1A shows that a host of EMT transcriptional regulators, including Twist, Snail and Slug, are increased in ECS cells (spheroid) as compared to non-stem cancer cells (monolayer). This is associated with increased levels of vimentin, fibronectin and N-cadherin, which are mesenchymal proteins, and reduced expression of E-cadherin, an epithelial marker. HIF-1α, an additional marker frequently associated with EMT, is also elevated. We next examined whether TG2 is required to maintain EMT marker expression. SCC-13 cell-derived ECS cells were grown in the presence of control- or TG2-siRNA, to reduce TG2, and the impact on EMT marker level was measured. Fig. 1B shows that loss of TG2 is associated with reduced expression of Twist, Snail, vimentin and HIF-1α. To further assess the role of TG2, we utilized SCC13-Control-shRNA and SCC13-TG2-shRNA2 cell lines. These lines were produced by infection of SCC-13 cells with lentiviruses encoding control- or TG2-specific shRNA. Fig. 1C shows that SCC13-TG2-shRNA2 cells express markedly reduced levels of TG2 and that this is associated with reduced expression of EMT associated transcription factors and target proteins, and increased expression of E-cadherin. To confirm this, we grew SCC13-Control-shRNA and SCC13-TG2-shRNA2 cells as monolayer cultures for immunostain detection of EMT markers. As shown in Fig. 2A, TG2 levels are reduced in TG2-shRNA expressing cells, and this is associated with the anticipated changes in epithelial and mesenchymal marker expression.

Tumor cells that express EMT markers display enhanced migration and invasion ability [24–26]. We therefore examined the impact of TG2 reduction on these responses. To measure invasion, control-shRNA and TG2-shRNA cells were monitored for ability to move through matrigel. Fig. 2B shows that loss of TG2 reduces movement through matrigel by 50%. We further show that this is associated with a reduction in cell migration using a monolayer culture wound closure assay. The control cells close the wound completely within 14 h, while TG2 knockdown reduces closure rate (Fig. 2C).

### TG2 inhibitor reduces EMT marker expression and EMT functional responses

**Figure 3:**
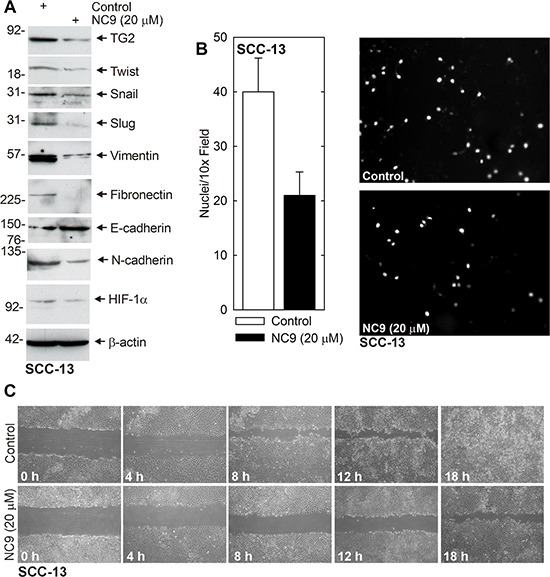
NC9 reduces EMT protein expression and invasion/migration **A.** SCC-13 cells (500,000) were plated in monolayer culture in spheroid medium in 100 mm dishes. Fresh spheroid medium containing 0 or 20 μM NC9 was added at time zero and again at 24 h. After 48 h lysates were collected for electrophoresis and immunoblot. **B.** SCC-13 cells were pretreated with 0 or 20 μM NC9 for 2 h and then harvested and plated (25,000 cells) in the upper chamber in 1 ml of medium, above a matrigel-coated membrane, in a Millicell chamber. After 30 h, the inside of membrane was harvested and the nuclei of migrated cells on the bottom of the membrane were counted using an inverted fluorescent microscope. The values are mean ± SEM, *n* = 3 (*p*, 0.05). **C.** SCC-13 (2 million) cells were plated in spheroid medium in 100 mm dishes and grown as monolayer cultures. Confluent monolayers were then scratched with a 10 μl pipette tip and wound width was monitored for 0–18 h. Similar results were observed in each of three experiments.

NC9 is a recently developed TG2-specific inhibitor [27, 28]. We therefore asked whether pharmacologic inhibition of TG2 suppresses EMT. SCC-13 cells were treated with 0 or 20 μM NC9. Fig. 3A shows that NC9 treatment reduces EMT transcription factor (Twist, Snail, Slug) and EMT marker (vimentin, fibronectin, N-cadherin, HIF-1α) levels. Consistent with these changes, the level of the epithelial marker, E-cadherin, is elevated. Fig. 3B and 3C show that pharmacologic inhibition of TG2 activity also reduces EMT biological response. Invasion (Fig. 3B) and cell migration (Fig. 3C) are also reduced.

### Identification of TG2 functional domain required for EMT

**Figure 4:**
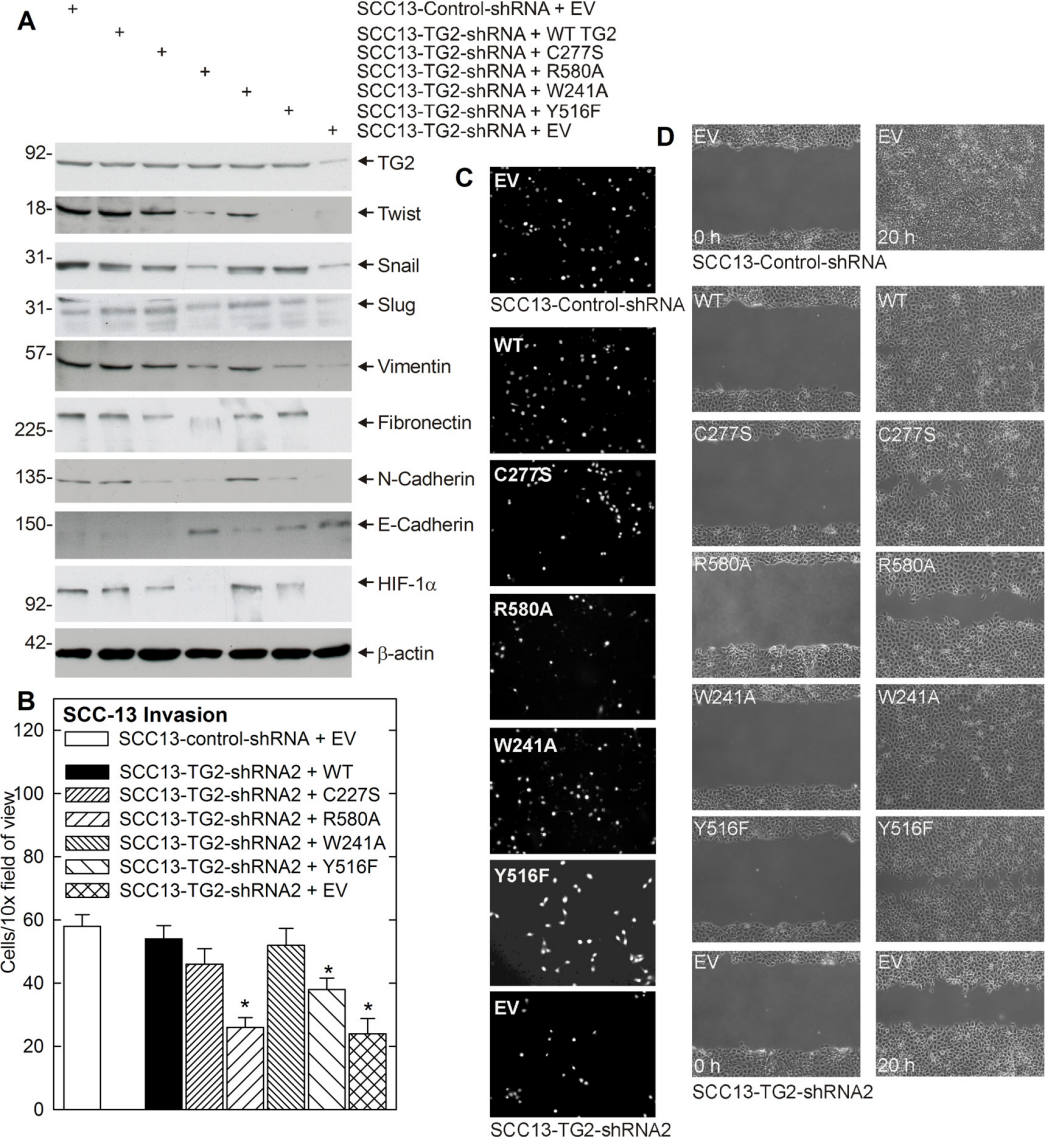
TG2 GTP binding/G-protein function is necessary for EMT **A.** TG2 impact on EMT marker expression. SSC13-Control-shRNA or SCC13-TG2-shRNA2 cells were electroporated with 3 μg of empty vector (EV) or vector encoding the indicated TG2 proteins. The cells were then grown as monolayer cultures in spheroid medium for three days. The cells were then harvested and extracts prepared for electrophoresis and immunoblot detection of the indicated epitopes. Similar results were observed in three experiments. **B.** SCC13-TG2-shRNA2 cells were electroporated with plasmids encoding wild-type TG2, mutant TG2, or empty vector and grown in spheroid medium as monolayers. At 48 h post-electroporation, SCC13-Control-shRNA and SCC13-TG2-shRNA2 cells were harvested and seeded in the upper chamber above a matrigel-coated membrane in 1 ml of spheroid medium containing 1% serum in a Millicell chamber. The lower chamber included spheroid medium containing 10% serum. Nuclei of migrated cells were stained and counted on the lower surface of the membrane using an inverted fluorescent microscope. The values are mean ± SEM, *n* = 3 (*p* < 0.01). **C.** Images of DAPI-stained membrane. **D.** SCC13-Control-shRNA and SCC13-TG2-shRNA2 cells were electroporated with empty vector (EV) or vector encoding the indicated TG2 proteins and grown in spheroid medium as monolayers. After 24 h, 2 million cells were plated in spheroid media on 100 mm dishes in monolayer conditions. The confluent monolayer cultures were then scratched with a 10 μl pipette tip and width of the wound was monitored at 0–18 h to assess closure. Similar results were observed in each of three experiments.

We next performed studies to identify the functional domains and activities required for TG2 regulation of EMT. TG2 is a multifunctional enzyme that serves as a scaffolding protein, as a transamidase, as a kinase, and as a GTP binding protein [21]. The two best studied functions are the transamidase and GTP binding/G-protein related activities [21]. Transamidase activity is observed in the presence of elevated intracellular calcium, while GTP binding-related signaling is favored by low calcium conditions (reviewed in [21]). To identify the TG2 activity required for EMT, we measured the ability of wild-type and mutant TG2 to restore EMT in SCC13-TG2-shRNA2 cells, which have reduced TG2 expression (Fig. 4A). SCC13-TG2-shRNA2 cells display reduced expression of EMT markers including Twist, Snail, Slug, vimentin, fibronectin, N-cadherin and HIF-1α, and increased expression of the epithelial maker, E-cadherin, as compared to SCC13-Control-shRNA cells. Expression of wild-type TG2, or the TG2-C277S or TG2-W241A mutants, restores marker expression in SCC13-TG2-shRNA2 cells (Fig. 4A). TG2-C277S and TG2-W241A lack transamidase activity [10, 29–31]. In contrast, TG2-R580A, which lacks G-protein activity [29–31], and TG2-Y516F, which retains only partial G-protein activity [30], do not efficiently restore marker expression. These findings suggest that the TG2 GTP binding function is required for EMT.

We next assayed the ability of the TG2 mutants to restore EMT functional responses - invasion and migration. Fig. 4B and 4C shows that wild-type TG2, TG2-C277S and TG2-W241A restore the ability of SCC13-TG2-shRNA2 cells to invade matrigel, but TG2-R580A and Y516F are less active. Fig. 4D shows a similar finding for cell migration, in that the TG2-R580A and Y517F mutant are only partially able to restore SCC13-TG2-shRNA2 cell migration. These findings suggest that TG2 GTP binding/G-protein related activity is required for EMT-related migration and invasion by skin cancer cells.

### Role of TG2 in regulating EMT in A431 cells

**Figure 5:**
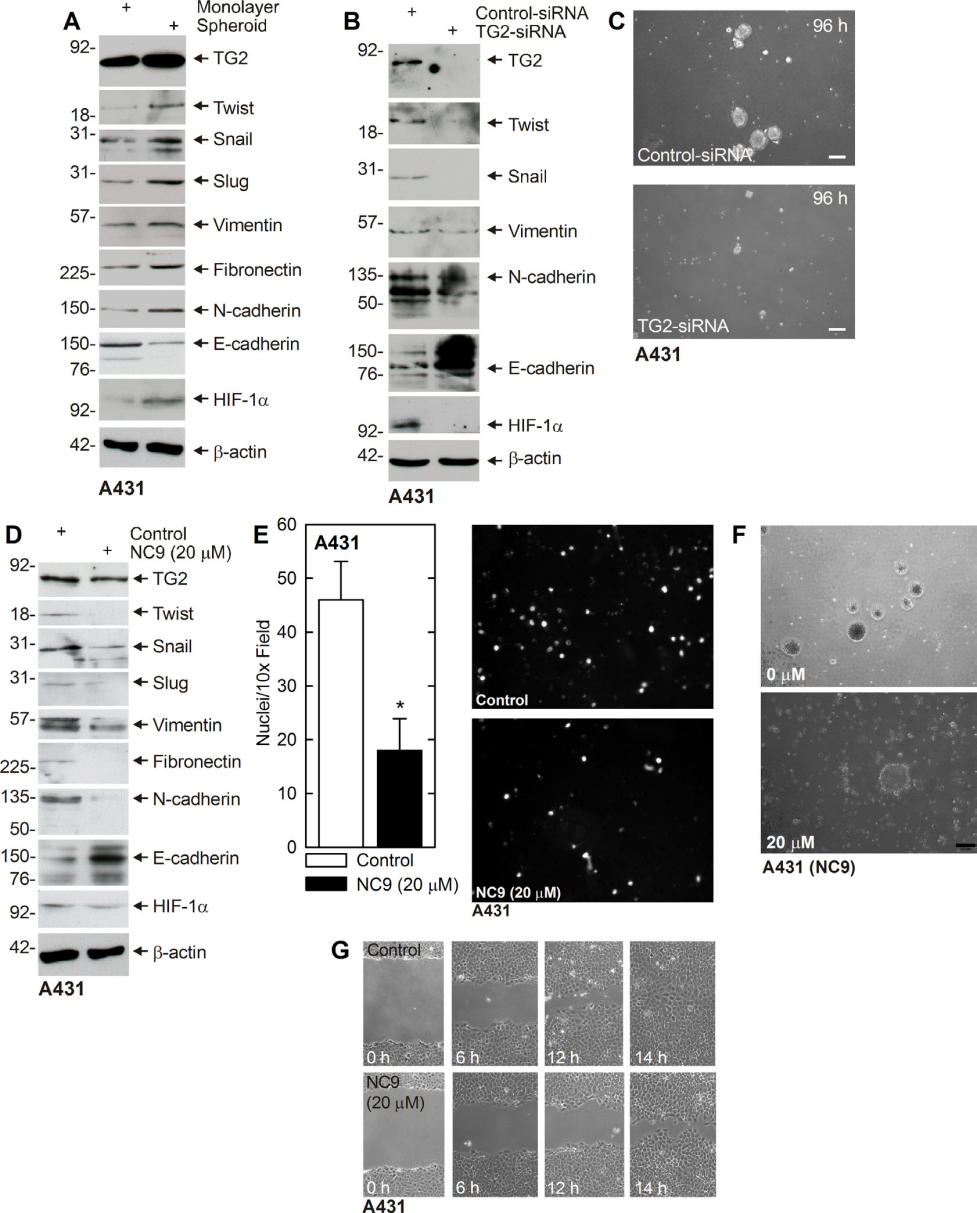
TG2, EMT markers and EMT response in A431 cells **A.** A431-derived ECS cells have elevated TG2 and mesenchymal markers. A431 cells (40,000 cells/well) were grown in spheroid media in monolayer conditions or as non-attached spheroids (ECS cells) for 10 d. Cells were harvested and lysates prepared for immunoblot. **B.** A431 cells were electroporated with 3 μg of control- or TG2-siRNA and at 72 h extracts were prepared to assay TG2 and EMT marker level. **C.** A431-derived ECS cells were electroporated with 3 μg of the indicated siRNA and then grown as spheroids for 96 h. Similar results were observed in three separate experiments. **D.** A431 cells (500,000 cells/well) were plated in monolayer culture in spheroid media in 100 mm dishes. The following day, at time zero, 0 or 20 μM NC9 was added with fresh addition of medim and NC9 at 24 h. At 48 h the cells were harvested and extracts prepared for immunoblot. **E.** A431 cells were pretreated in 0 or 20 μM NC9 for 2 h and then harvested and 25,000 cells were seeded in the upper chamber above a matrigel-coated membrane in 1 ml of growth medium containing 1% FCS in a Millicell chamber. The lower chamber was filled with growth medium containing 10% FCS. After 30 h, the membrane was removed, washed and stained with DAPI, and an inverted fluorescent microscope was used to count the nuclei of migrated cells. **F.** A431 (40,000) cells were plated in spheroid media in non-attached conditions. On d 8, spheroids were treated with 0 or 20 μM NC9 and images were taken after 48 h to monitor spheroid fragmentation. **G.** A431 (2 million) cells were plated in spheroid media on 100 mm dishes and grown as monolayers. Confluent monolayers were then scratched with a 10 μl pipette tip, rinsed, and width of the wound was monitored for 0–14 h. Similar results were observed in each of three experiments.

The number of available epidermis-derived squamous cell carcinoma cell lines is limited, and so we compared our findings with A431 cells. A431 cells are squamous cell carcinoma cells established from human vulvar skin. A431 cells were grown as monolayer (non-stem cancer cells) and spheroids (ECS cells) and after 10 d the cells were harvested and assayed for expression of TG2 and EMT makers. Fig. 5A shows that TG2 levels are elevated in ECS cells and that this is associated with increased levels of mesenchymal markers, including Twist, Snail, Slug, vimentin, fibronectin, N-cadherin and HIF-1α. In contrast, E-cadherin levels are reduced. We next examined the impact of TG2 knockdown on EMT marker expression. Fig. 5B shows that mesenchymal markers are globally reduced and E-cadherin level is increased. As a biological endpoint of EMT, we examine the impact of TG2 knockdown on spheroid formation and found that TG2 loss leads to reduced spheroid formation (Fig. 5C). We next examined the impact of NC9 treatment on EMT and found a reduction in EMT markers expression associated with an increase in epithelial (E-cadherin) marker level (Fig. 5D). This loss of EMT marker expression is associated with reduced matrigel invasion (Fig. 5E), reduced spheroid formation (Fig. 5F) and reduced cell migration (Fig. 5G).

### Role of NFκB

**Figure 6:**
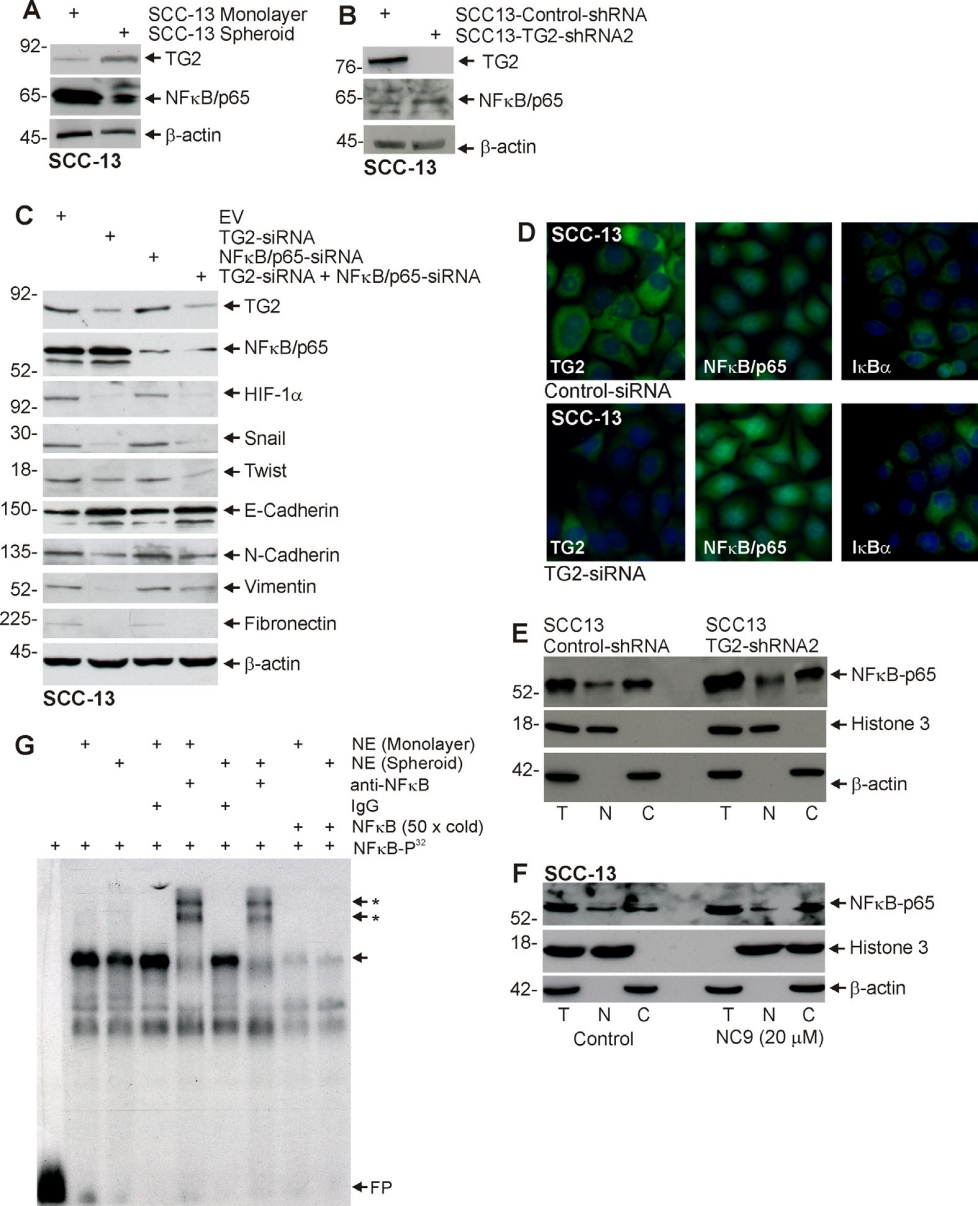
NFκB expression is not required for EMT **A.** ECS cells have reduced NFκB-p65 levels. SCC-13 cells (40,000) were grown in spheroid medium in attached (monolayer) and non-attached (spheroid, ECS cells) conditions. Lysates were prepared after 10 d in culture for electrophoresis and detection of indicated epitopes. **B.** SCC13-Control-shRNA and SCC13-TG2-shRNA2 cells were grown in spheroid medium as monolayers. After 10 d in culture, lysates were collected for detection of TG2 and NFκB-p65. **C.** SCC-13 cells were electroporated with control-, NFκB-p65- or TG2-siRNA and then cultured as monolayers in spheroid medium. After 72 h extracts were prepared to assay TG2, NFκB-p65 and EMT markers. **D.** SCC-13 cells were electroporated with empty vector Control- or TG2-siRNA and plated in monolayer culture. The following day cells were fixed, permeabilized and incubated with antibodies specific for the indicated epitopes. Similar results were observed in each of three experiments. **E.** An equal number of cell equivalents of total (T), nuclear (N), and cytosolic (C) extract, prepared from SCC13-Control-shRNA and SCC13-TG2-shRNA2 (TG2 knockdown) cells, was electrophoresed for immunoblot detection of NFκB, histone 3 (nuclear marker) and β-actin (cytoplasmic marker). Similar results were observed in three separate experiments. **F.** Total (T), nuclear (N), and cytosolic (C) extract was prepared from 8 day spheroids (ECS cells) treated with 0 or 20 μM NC9 for 48 h. An equal number of cell equivalents of total, nuclear and cytosol extract was electrophoresed for immunoblot detection with the indicated antibodies. Similar results were observed in three separate experiments. **G.** Nuclear extract (NE), prepared from non-stem cancer cells (monolayer) or ECS cells (spheroids), was incubated with ^32^P-NFκB probe and electrophoresed on a non-denaturing 6% acrylamide gel. Various groups were incubated with IgG, anti-NFkB or a 50-fold (50× cold) molar excess of radioinert NFkB probe prior to electrophoresis. FP indicates free ^32^P-NFκB probe, the arrow indicates the gel mobility shift and the arrows with asterisks indicate the anti-dependent supershifted bands.

Previous studies in breast [18, 32–36], ovarian cancer [12, 37, 38], and epidermoid carcinoma [11] indicate that NFκB signaling mediates TG2 impact on EMT. We therefore assessed the role of NFκB in skin cancer cells. As shown in Fig. 6A, the increase in TG2 level observed in ECS cells (spheroids) is associated with reduced NFκB level. In addition, NFκB level is increased in TG2 knockdown cells (Fig. 6B). Thus, increased NFκB is not associated with increased TG2. We next assessed the impact of NFκB knockdown on TG2 control of EMT marker expression. Fig. 6C shows that TG2 is required for increased expression of EMT markers (HIF-1α, snail, twist, N-cadherin, vimentin and fibronectin) and reduced expression of the E-cadherin epithelial marker; however, knockdown of NFκB expression does not interfere with TG2 regulation of these endpoints. We next examined the effect of TG2 knockdown on NFκB and IκBα localization. The fluorescence images in Fig. 6D suggest that TG2 knockdown with TG2-siRNA does not alter the intracellular localization of NFκB or IκBα. This is confirmed by subcellular fractionation assay (Fig. 6E) which compares NFκB level in SCC13-TG2-Control and SCC13-TG2-shRNA2 (TG2 knockdown) cells. We also monitored NFκB subcellular distribution following treatment with NC9, the TG2 inhibitor. Fig. 6F shows that cytoplasmic/nuclear distribution of NFκB is not altered by NC9. Finally, we monitored the impact of TG2 expression on NFκB binding to a canonical NFκB-response element. Increased NFκB binding to the response element is a direct measure of NFκB activity [10]. Fig. 6G shows that overall binding is reduced in nuclear (N) extract prepared from ECS cells (spheroids) as compared to non-stem cancer cells (monolayer), and that NFkB binding, as indicated by gel supershift assay, is also slightly reduced in ECS cell extracts. These findings indicate that NFkB binding is slightly reduced in ECS cells, which are TG2-enriched (Fig. 1A).

**Figure 7:**
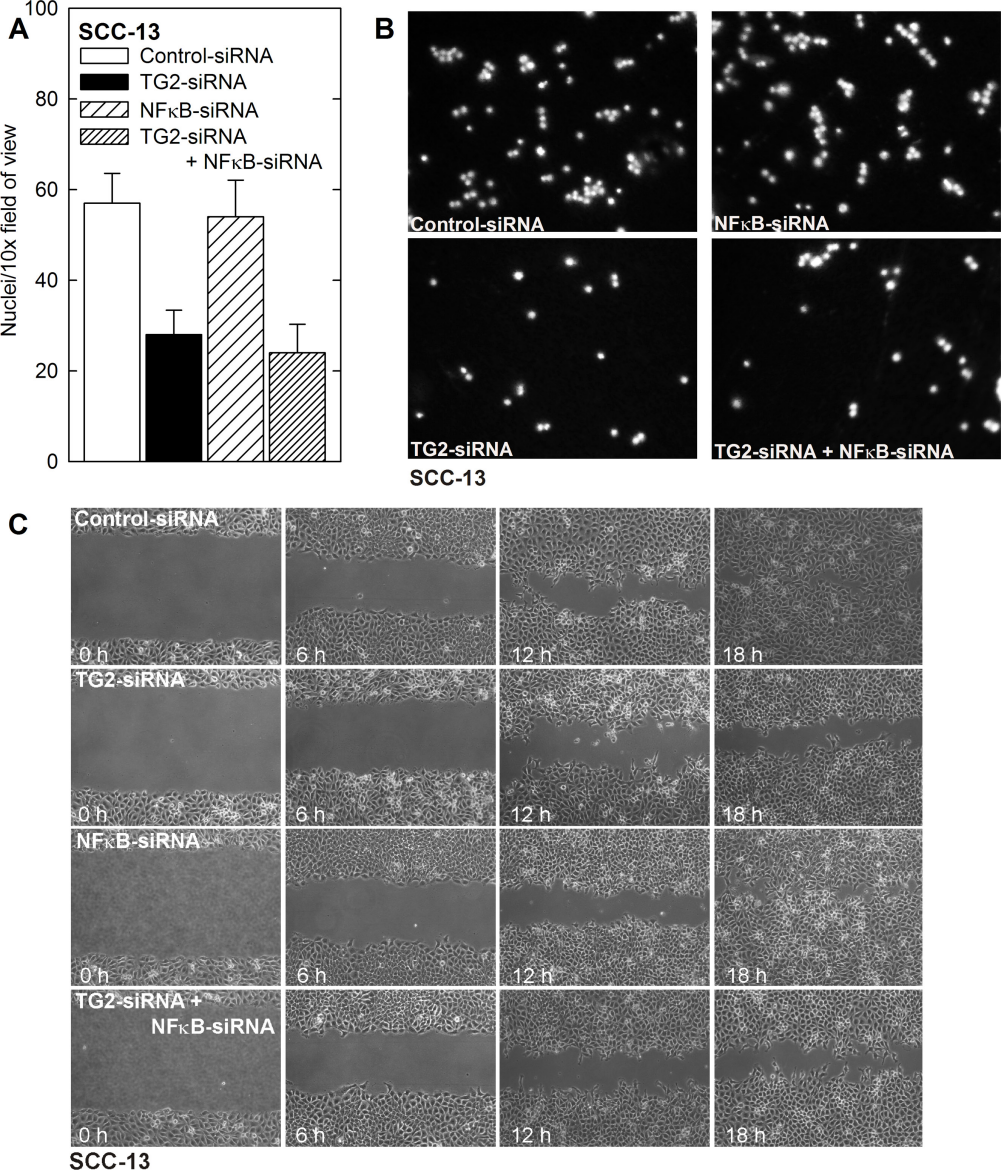
NFκB is not required for ECS cell migration and invasion **A, B.** NFκB-knockdown does not suppress ECS cell matrigel invasion. SCC-13 cells were treated with Control-, TG2-, NFκB- or a combination of siRNA and then seeded in the upper chamber above a matrigel-coated membrane in 1 ml of growth medium containing 1% FCS in a Millicell chamber. The lower chamber was filled with growth medium containing 10% FCS. After 30 h, the membrane was removed, washed and stained with DAPI, and an inverted fluorescent microscope was used to count the nuclei of migrated cells. **C.** NFκB-knockdown does not impede ECS cell migration. SCC-13 cells (2 million) cells were plated in spheroid media on 100 mm dishes and grown as monolayers. Confluent monolayers were then scratched with a 10 μl pipette tip, rinsed, and width of the wound was monitored for 0–18 h. Similar results were observed in each of three experiments.

We next monitored the role of NFκB on biological endpoints of EMT. Fig. 7A and 7B show that TG2 knockdown reduces migration through matrigel, but NFκB knockdown has no impact. Likewise, TG2 knockdown reduces wound closure, but NFκB knockdown does not. These findings suggest that NFκB does not mediate the pro-EMT actions of TG2 in epidermal squamous cell carcinoma.

## DISCUSSION

The metastatic cascade, from primary tumor to metastasis, is a complex process involving multiple pathways and signaling cascades [39–41]. Cells that complete the metastatic cascade migrate away from the primary tumor through the blood to a distant site and there form a secondary tumor. Identifying the mechanisms that allow cells to survive this journey and form secondary tumors is an important goal. The processes involved in epithelial-mesenchymal transition (EMT) are important cancer therapy targets, as EMT is associated with enhanced cancer cell migration and stem cell self-renewal. EMT regulators, including Snail, Twist, Slug, are increased in expression in EMT and control expression of genes associated with the EMT phenotype [42].

### TG2 is required for EMT

We have characterized a population of ECS cells derived from epidermal squamous cell carcinoma [3]. The present studies show that these cells, which display enhanced migration and invasion, possess elevated levels of TG2. Moreover, these cells are enriched in expression of transcription factors associated with EMT (Snail, Slug, and Twist, HIF-1α) as well as mesenchymal structural proteins including vimentin, fibronectin and N-cadherin. Consistent with a shift to mesenchymal phenotype, E-cadherin, an epithelial marker, is reduced in level. Additional studies show that TG2 knockdown results in a marked reduction in EMT marker expression and that this is associated with reduced ability of the cells to migrate to close a scratch wound and reduced movement in matrigel invasion assays. We also examined the impact of treatment with a TG2 inhibitor. NC9 is an irreversible active site inhibitor of TG2, that locks the enzyme in an open conformation [28, 43–45]. NC9 treatment of ECS cells results in decreased levels of Snail, Slug and Twist. These transcription factors suppress E-cadherin expression [46] and their decline in level is associated with increased levels of E-cadherin. NC9 inhibition of TG2 also reduces expression of vimentin, fibronectin and N-cadherin, and these changes are associated with reduced cell migration and reduced invasion through matrigel.

We also examined the role of TG2 in A431 squamous cell carcinoma cells derived from the vulva epithelium. TG2 is elevated in A431-derived ECS cells, as are EMT markers, and knockdown of TG2, with TG2-siRNA, reduces EMT marker expression and spheroid formation. Studies with NC9 indicate that NC9 inhibits A431 spheroid formation, EMT, migration and invasion. These studies indicate that TG2 is also required for EMT and migration and invasion in A431 cells. Based on these findings we conclude that TG2 is essential for EMT, migration and invasion, and is likely to contribute to metastasis in squamous cell carcinoma.

### TG2 GTP binding activity is required for EMT

TG2 is a multifunctional enzyme that can act as a transamidase, GTP binding protein, protein disulfide isomerase, protein kinase, protein scaffold, and DNA hydrolase [21, 29, 44, 47]. The two most studied functions are the transamidase and GTP binding functions [29, 44, 47]. To identify the TG2 activity responsible for induction of EMT, we studied the ability of TG2 mutants to restore EMT in SCC13-TG2-shRNA2 cells, which express low levels of TG2 and do not express elevated levels of EMT markers or display EMT-related biological responses. These studies show that wild-type TG2 restores EMT marker expression and the ability of the cells to migrate on plastic and invade matrigel. TG2 mutants that retain GTP binding activity (TG2-C277S and TG2-W241A) also restore EMT. In contrast, TG2-R580A, which lacks GTP binding function, does not restore EMT. This evidence suggests that the GTP binding function is essential for TG2 induction of the EMT phenotype in ECS cells. Recent reports suggest that the TG2 is important for maintenance of stem cell survival in breast [9, 10, 17] and ovarian [12, 38, 48] cancer cells. Moreover, our findings are in agreement of those of Mehta and colleagues who reported that the TG2 GTP binding function, but not the crosslinking function, is required for TG2 induction of EMT in breast cancer cells [10].

### TG2, NFκB signaling and EMT

To gain further insight into the mechanism of TG2 mediated EMT, we examined the role of NFκB. NFκB has been implicated as mediating EMT in breast, ovarian, and pancreatic cancer; however, NFκB may have a unique role in epidermal squamous cell carcinoma. In keratinocytes, NFκB has been implicated in keratinocyte dysplasia and hyperproliferation [49]. However, inhibition of NFκB function has also been shown to predispose murine epidermis to cancer [50]. Here we show that TG2 levels are elevated and NFκB levels are reduced in ECS cells as compared to non-stem cancer cells, and that TG2 knockdown is associated with increased NFκB level. In addition, TG2 knockdown, or inhibition of TG2 by treatment with NC9, does not altered the nuclear/cytoplasmic distribution of NFκB. We further show that elevated levels of TG2 in spheroid culture results in a slight reduction in NFκB binding to the NFκB response element, as measured by gel mobility supershift assay. These molecular assays strongly suggest that NFκB does not mediate the action of TG2 in epidermal cancer stem cells. Moreover, knockdown of NFκB-p65 in TG2 positive cells does not result in a reduction in Snail, Slug and Twist, or mesenchymal marker proteins expression, and concurrent knockdown of TG2 and NFκB does not reduce EMT marker protein levels beyond that of TG2 knockdown alone. These findings suggest that NFκB is not an intermediary in TG2-stimulated EMT in ECS cells. This is in contrast to the required role of NFκB in mediating TG2 induction of cell survival and EMT in breast cancer cells [18, 32, 33] and ovarian cancer [12, 37, 38] and epidermoid carcinoma [11].

## MATERIALS AND METHODS

### Reagents

Cells were maintained as monolayer cultures in growth medium including DMEM (Invitrogen, Frederick, MD) supplemented with 4.5 mg/ml D-glucose, 200 mM L-glutamine, 100 mg/ml sodium pyruvate, and 5% fetal calf serum. Heat-inactivated fetal calf serum (FCS, F4135) and anti-β-actin (A5441) were purchased from Sigma (St. Louis, Mo). Cell lysis Buffer (9803) was purchased from Cell Signaling Technology (Danvers, MA). Anti-TG2 (2187378) was purchased from EMD Millipore (Bedford, MA). Antibodies for HIF-1α (ab113642), Twist (ab49254) and Slug (ab27568) were purchased from Abcam. Antibodies for vimentin (5741) and Snail (3895) were purchased from Cell Signaling Technologies. NFκB-p65 antibody (sc-109) was purchased from Santa Cruz (Santa Cruz, CA). Anti-E-cadherin was purchased from Epitomics (Ab40772). N-cadherin (610920) and fibronectin (610077) antibodies were purchased from BD Biosciences (San Jose, CA). Peroxidase-conjugated anti-mouse IgG (NXA931) and anti-rabbit IgG (NA934V) were obtained from GE Healthcare (Buckinghamshire, UK). Alexa Fluor 555 goat anti-mouse IgG (A21424) and Alexa Fluor 488 goat-anti-rabbit IgG (A11034) were purchased from Invitrogen. DAPI (D9542) was purchased from Sigma. NC9 synthesis was previously described [27, 28]. TG2- (sc-37514) and control-siRNA (sc-37007) were purchased from Santa Cruz (Dallas, TX). Matrigel (354234) and BD Biocoat cell inserts (353097) were purchased from BD Biosciences.

### Immunoblot

Equivalent amounts of protein were electrophoresed on denaturing and reducing 10% polyacrylamide gels and transferred to nitrocellulose membrane. The membrane was blocked by 5% nonfat dry milk for 1 h and incubated with primary antibody (diluted 1:1000) in 5% nonfat dry milk. Blots were rinsed and then incubated with secondary antibody (diluted 1:5000) for 2 h. Secondary antibody binding was visualized using ECL Prime chemiluminescence detection technology (Amersham).

### TG2-shRNA lentivirus production

TG2-shRNA encoding lentivirus was produced using 293T packaging cells maintained in Dulbecco's modified Eagle's medium containing 1 mM L-glutamine, 1 mM sodium pyruvate and 10% fetal calf serum. Cells were plated in 100 mm dishes at 50% confluence 24 h prior to transfection. The media was removed, and the cells washed with Hank's Balanced Salt Solution and transferred to serum-free growth medium 24 h before prior to transfection with 1 μg of pCMV-VSVG, 0.5 μg pCMV-dr8.91 and 0.5 μg TG2-shRNA encoding plasmid. pCMV-VSVG (8454) and pCMV-dr8.91 were purchased from Addgene and kindly provided by Dr. CY Lin. The lentivirus plasmids, pLKO.1-NT-Puro-shRNA (Control) (SHC016) and pLKO.1-Puro-hTGM2-shRNA (TRCN-0000272760) were from Sigma-Aldrich (St. Louis, MO). At 3 h post-transfection, fresh medium containing 10% FCS was added. After an additional 72 h the conditioned-medium was collected, centrifuged for 15 min at 1,500 RPM, filtered through a 22 μm filter and pipetted into aliquots for storage at −80 C.

### Stable TG2 knockdown cell lines

SCC-13 cells (1 × 10^5^) were plated in 24 well cluster plates and allowed to attach overnight, followed by incubation with 1 ml of pLKO.1-Puro-hTGM2-shRNA lentivirus in serum-free growth media containing 8 μg/ml polybrene at 37 C for 5 h. The media was then supplemented with 5% fetal calf serum followed by selection for two weeks with 0.25 μg/ml puromycin. The resulting cells were infected a second time with the same virus and reselected with puromycin to produce the SCC13-TG2-shRNA2 cell line. TG2 knockdown was confirmed by anti-TG2 immunoblot. A control cell line, SCC13-Control-shRNA, was produced by double infection with pLKO.1-Puro-NT-shRNA lentivirus, which encodes control-shRNA, using an identical protocol.

### Spheroid formation assay

Cells, maintained as monolayer cultures in growth medium consisting of DMEM (Invitrogen, Frederick, MD) supplemented with 4.5 mg/ml D-glucose, 200 mM L-glutamine, 100 mg/ml sodium pyruvate, and 5% fetal calf serum, were harvested, collected by centrifugation, and resuspended in spheroid medium comprising DMEM/F12 (1:1) (DMT-10-090-CV, Mediatech INC, Manassa, VA) supplemented with 2% B27 serum-free supplement (17504-044, Invitrogen, Frederick, MD), 20 ng/ml EGF (E4269, Sigma, St. Louis), 0.4% bovine serum albumin (B4287, Sigma) and 4 μg/ml insulin (19278 Sigma, St. Louis, MO.). The cells (40,000) were plated in 9.6 cm^2^ wells in Costar six well ultra-low attachment cluster dishes (4371, Corning, Tewksbury, MA). Spheroid size and number was monitored with time in culture as previously described [3].

### Electroporation of nucleic acids

Cells were maintained in monolayer culture in growth medium. Near-confluent cultures were harvested the day prior to electroporation and plated in 60 mm dishes in growth medium. After 24 h, when 50% confluent, the cells were re-trypsinized, centrifuged at 200 × g, and replated. The next morning 1 × 10^6^ cells were harvested, washed with sterile phosphate-buffered saline, suspended in 100 μl of keratinocyte nucleofection reagent containing 3 μg of plasmid or siRNA and electroporated using the Amaxa Electroporator on the T-018 setting [51]. Immediately after electroporation, the cells were resuspended in pre-warmed medium and plated. When siRNA was used, the cells were harvested at 72 h post-electroporation and re-electroporated a second time. This double electroporation assured sustained target knockdown.

### Invasion assay

Matrigel was diluted in 0.01 M Tris-HCl/0.7% NaCl, filter sterilized and 0.1 ml was used to coat individual BD BioCoat inserts (Millicell-PCF, 0.4 μm, 12 mm, PIHP01250). Cells (25,000) were plated in 100 μl in growth medium, supplemented with 1% FCS, in the upper chamber. The lower chamber contained growth medium supplemented with 10% FCS. After migration, the membranes were harvested and excess cells were removed from the upper membrane surface. The membrane was fixed in 4% paraformaldehyde, stained with 1 μg/ml DAPI, and the underside of the membrane was photographed with an inverted fluorescent microscope and the number of cells counted.

### Migration assay

SCC13-Control-shRNA or SCC13-TG2-shRNA2 cells (2 × 10^6^) were plated in 10 cm dishes and grown as monolayer cultures in spheroid medium until confluent. A 10 μl pipette tip was used to prepare areas void of cells and the dishes were washed to remove the dislodged cells. Images were collected, at 0–18 h after the scratch using the 10× objective, and the width of the opening was measured as a function of time as an index of cell migration potential.

### Gel mobility shift assay

For gel mobility shift assay, 3 μg of nuclear extract was incubated for 30 min at room temperature in a 20 μl volume containing 20 mM HEPES, pH 7.5, 10% glycerol, 50 mM KCl, 2 mM MgCl_2_, 0.5 mM EDTA, 0.5 mM DTT, 1 mg/ml poly(dI:dC), 0.1 mg/ml bovine serum albumin, and 40,000 cpm radioactive double-stranded ^32^P-NFκB binding site oligonucleotide (5′-AGTTGAGGGGACTTTCCCAGGC). For competition studies, a 50-fold molar excess of non-radioactive competitor NFκB oligonucleotide was added to the DNA binding reaction. For gel mobility supershift assay, 2 μg of normal rabbit IgG (sc-3888) or rabbit anti-NFκB (sc-109), purchased from Santa Cruz Biotechnology, was added to the reaction mixture and incubated 1 h at 25 C. The ^32^P-labeled probe was then added and the incubation was continued for an additional 30 min at 25 C. Protein-DNA complexes were resolved by electrophoresis on a 6% polyacrylamide nondenaturing gel [52].

### Cell fractionation

Cell fractionation was performed using the NE-PER Nuclear and Cytoplasmic Extraction Kit (78440) and Halt protease inhibitor (78440) obtained from Thermo-Scientific (Waltham, MA). Four million cells were trypsinized, washed in phosphate-buffered saline and pelleted. The pellet was resuspended in 200 μl of ice cold CER I buffer and maintained for 10 min on ice. Ice-cold CER II buffer (50 μl) was added, the sample was vortexed and maintained on ice for 1 min prior to centrifugation. The supernatant (cytosol) was then collected as a total volume of 250 μl and stored at −80 C. The nuclear pellet was suspended, by repeated vortexing, in 50 μl of ice cold NER buffer over 40 min. The sample was then centrifuged for 10 min and the nuclear extract was stored at −80 C until use. For analysis, 0.56 million cell equivalents of nuclear (35 μl) or cytosolic (7 μl) extract was electrophoresed for immunoblot detection of NFκB, histone H3 and β-actin.
